# Krypton-85 datasets of the northern and southern hemisphere collected over the past 60 years

**DOI:** 10.1016/j.dib.2020.106522

**Published:** 2020-11-13

**Authors:** Arne Kersting, Clemens Schlosser, Sabine Schmid, Martina Konrad, Andreas Bollhöfer, Karen Barry, Axel Suckow

**Affiliations:** a Institute of Environmental Physics, Heidelberg University, 69120 Heidelberg, Germany; b Bundesamt für Strahlenschutz, Rosastr. 9, 79098 Freiburg, Germany; c CSIRO Land & Water, Gate 5 Waite Road, Urrbrae, SA 5064, Australia

**Keywords:** krypton-85, Tracer, Dating, Monitoring, Noble gas radionuclides

## Abstract

With a half-life of 10.7 years, the noble gas radioisotope ^85^Kr is perfectly suited as a tracer to date ice and water that formed during the past half century. Furthermore, due to its inhomogeneous input into the atmosphere, it is a useful tool to investigate atmospheric circulation and back-trajectory analysis. The data presented here represent a comprehensive time series of atmospheric ^85^Kr activity concentrations in ground level air that can be used to model northern and southern hemispheric input functions, which is essential to apply ^85^Kr as a dating tracer. The collection comprises 11 datasets from 4 monitoring stations in the northern and 7 monitoring stations in the southern hemisphere, respectively. In total, it contains about 8000 measurements performed over the past 60 years, making it the largest published ^85^Kr record.

## Specifications Table

**Table:** 

Subject	Atmospheric Science
Specific subject area	Monitoring of the atmospheric ^85^Kr concentrations in ground level air in the northern and southern hemisphere
Type of data	Excel file in data repository, Table Figure
How data were acquired	The ^85^Kr activity concentration data were acquired after gaschromatographic separation of krypton followed by β-decay counting of Kr-85 in gas proportional counters.
Data format	Raw
Parameters for data collection	The krypton was sampled from ground level air and the monitoring stations were carefully chosen, to avoid contamination with locally produced ^85^Kr.
Description of data collection	Primary data of the data collection comprises the Adelaide dataset and all samples of the Schauinsland, Jungfraujoch and Freiburg datasets taken since (December 2018). All other data is secondary data taken from the cited papers.
Data source location	Primary data sources: Adelaide: Institution: CSIRO City/Town/Region: Adelaide Country: South Australia Lat/Lon of monitoring station: 34°58’ S 138°38’ E Antarctica: Institution: Institute of Environmental Physics, University Heidelberg, Germany City/Town/Region: Georg von Neumayer Station Country: Antarctica Lat/Lon of monitoring station: 70°40’ S 08°16’ W Cape Grim: Institution: CSIRO & Institute of Environmental Physics, University Heidelberg, Germany City/Town/Region: Tasmania Country: Australia Lat/Lon of monitoring station: 40°41’ S 144°41’ E Cape Point: Institution: Institute of Environmental Physics, University Heidelberg, Germany City/Town/Region: Cape of Good Hope Country: South Africa Lat/Lon of monitoring station: 34°32’ S 18°29’ E Darwin: Institution: Supervising Scientist Division, Environment Australia City/Town/Region: Darwin Country: Australia Lat/Lon of monitoring station: 12°28’ S 130°50’ E Early Measurements: Institutions: Physics Department, University Heidelberg, Germany Institute of Environmental Physics, University Heidelberg, Germany Planck Institute for Nuclear Physics, Heidelberg, Germany Max Planck Institute for Nuclear Physics, Freiburg-Schauinsland Branch, Germany Institute for Atmospheric Radioactivity (IAR), Federal Office of Civil Defence, Freiburg, Germany Commissariat a l'Energie Atomique, Department de la Protection Sanitaire, Fontenay-aux-Roses, France Air Resources Laboratories, National Oceanic and Atmospheric Administration, Silver Spring, MD 20910, U.S.A Freiburg: Institution: Bundesamt für Strahlenschutz City/Town/Region: Freiburg Country: Germany Lat/Lon of monitoring station: 48°00’ N 07°51’ E Jungfraujoch: Institution: Bundesamt für Strahlenschutz City/Town/Region: Berner Alps
	Country: Swiss Lat/Lon of monitoring station: 46°24’ N 08°42’ E Schauinsland: Institution: Bundesamt für Strahlenschutz City/Town/Region: Freiburg Country: Germany Lat/Lon of monitoring station: 47°55’ N 07°54’ E Tahiti: Institution: Office of Atomic Energy City/Town/Region: Tahiti Country: French Polynesia Lat/Lon of monitoring station: 17°37’ S 149°28’ W Terre-Adélie: Institution: Office of Atomic Energy City/Town/Region: Eastern Antarctica Country: Antarctica Lat/Lon of monitoring station: 66°40’ S 140°00’ E
Data accessibility	Kersting, Arne; Bollhöfer, Andreas; Schlosser, Clemens; Schmid, Sabine; Konrad, Martina; Barry, Karen; Suckow, Axel (2020), “Atmospheric krypton-85 activity concentrations”, Mendeley Data, V1, doi:10.17632/p32bmw6rgs.1 Direct URL to data: https://data.mendeley.com/datasets/p32bmw6rgs/1
Related research article	Kersting, C. Schlosser, A. Bollhöfer und A. Suckow, “Evaluating 5 decades of atmospheric 85Kr measurements in the southern hemisphere to derive an input function for dating water and ice with implications for interhemispheric circulation and the global 85Kr emission inventory.“ Journal of Environmental Radioactivity. In Press https://doi.org/10.1016/j.jenvrad.2020.106451

## Value of the Data

• This comprehensive dataset is important for the application of ^85^Kr as a dating tracer in water and ice
• Researchers in the field of tracer hydrology can benefit from these data as it allows deriving a ^85^Kr input function for dating
• The ^85^Kr data is useful for investigating atmospheric circulation and it can support back trajectory models due to nuclear reprocessing plants as point like sources of ^85^Kr.
• The dataset will support the potential future application of ^85^Kr as a tool for the verification of nuclear arms control treaties.

## 1 Data Description

The data collection consists of 11 datasets of atmospheric ^85^Kr activity concentrations with 4 datasets from monitoring stations in the northern hemisphere ("Early Measurements NH", "Freiburg", "Schauinsland" and "Jungfraujoch") and 7 datasets from monitoring stations in the southern hemisphere ("Adelaide", "Antarctica", Cape Grim", "Cape Point", "Darwin", "Tahiti" and "Terre-Adelie") (Table 1).

**Table 1:** List of all the datasets with start and end of the sampling as well as the number of total samples and the elevation of the monitoring station.

Dataset	First sample	Last sample	Measurements	Elevation [m a.s.l.]
Adelaide	20.07.2015	17.06.2020*	255	140
Antarctica	08.06.1983	18.01.2008	415	20
Cape Grim [1]	14.04.1987	18.07.1996	166	40
Cape Point	21.08.1985	31.10.1997	415	240
Darwin [2]	15.08.2007	24.05.2010	120	10
Early Measurements NH [3,4,5,6,7,8,9,10,11,12]	13.12.1959	15.05.1976	204	-
Freiburg [13]	15.06.1973	13.07.2020*	2386	280
Jungfraujoch [13]	06.01.1990	15.06.2020*	1547	3500
Schauinsland [13]	03.02.1976	12.07.2020*	2386	1200
Tahiti [5, 14]	18.08.1969	15.11.1976	18	10
Terre-Adélie [5, 14]	17.03.1968	05.10.1977	32	10

⁎ At these monitoring stations, sampling is still ongoing. The date just refers to the last sample in the dataset.

All measurements were conducted via β-decay counting in gas proportional counters with a measurement uncertainty of about 3%. However, for the datasets "Tahiti" and "Terre-Adelie" no errors were given in the original publications and a conservative estimate of 10% measurement uncertainty was taken.

**Fig. 1:**
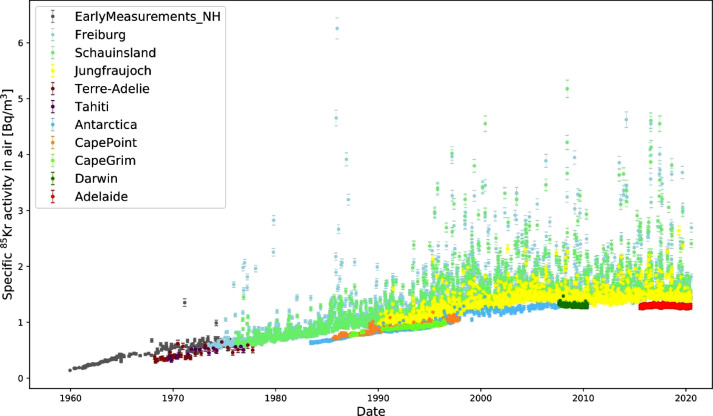
The ^85^Kr activity concentration in ground level air is plotted against the sampling date for all 11 datasets.

As seen in Fig. 1, the northern hemispheric data represents a coherent 60 years long series of measurements, while the southern hemispheric data set contains gaps of about 5 years between around 1980 and in the early 2010s. The ^85^Kr activity concentrations in the Freiburg, Schauinsland and Jungfraujoch dataset reach up to 6 Bq/m³ air, while the southern hemispheric data do not exceed 1.5 Bq/m³ air.

## 2 Experimental Design, Materials and Methods

The collection of krypton samples for the analysis of ^85^Kr follows the same principle for all datasets. In a multistage process, 2 to 5 ml of pure krypton are separated from about 10 m³ of air and the ^85^Kr activity concentration is determined via radioactive β-decay counting in gas proportional counters.

The first separation step is done by pumping air for one week with a constant flow of about 1 L/min through a liquid nitrogen cooled activated charcoal column. The pressure in the column is regulated to about 500 mbar to avoid condensation of oxygen and nitrogen, while most of the krypton is trapped [15]. After one week, the activated charcoal column is replaced with a clean column, to ensure continuous sampling. The charged column is heated to 300°C and the released gas is flushed into a 1 L aluminium container with helium as carrier gas. For the second purification step, the 1 L aluminium container is shipped to the laboratories of the “Bundesamt für Strahlenschutz” in Freiburg, Germany.

Via cryogenic purification, CO_2_ is removed, and the residual gas mixture is flushed with helium through a smaller liquid nitrogen cooled activated charcoal trap to further remove the lighter air components, mainly O_2_, N_2_ and Ar.

In a third step, krypton is separated from xenon by gas chromatography with methane serving as a carrier and counting gas. The highly enriched krypton fraction is then flushed into a gas proportional counter to measure its ^85^Kr activity.

The overall measurement uncertainty for an atmospheric ^85^Kr measurement is about 3% with a ^85^Kr detection limit of typically around 4 mBq/m³ air.

## CRediT authorship contribution statement

**Arne Kersting:** Data curation, Writing - original draft. **Clemens Schlosser:** Conceptualization, Investigation, Validation, Project administration. **Sabine Schmid:** Data curation, Validation. **Martina Konrad:** Data curation. **Andreas Bollhöfer:** Writing - review & editing, Supervision. **Karen Barry:** Data curation. **Axel Suckow:** Data curation, Writing - review & editing.

## Declaration of Competing Interest

The authors declare that they have no known competing financial interests or personal relationships which have or could be perceived to have influenced the work reported in this article.
